# Foliar Application of Wood Distillate Alleviates Ozone-Induced Damage in Lettuce (*Lactuca sativa* L.)

**DOI:** 10.3390/toxics10040178

**Published:** 2022-04-05

**Authors:** Andrea Vannini, Riccardo Fedeli, Massimo Guarnieri, Stefano Loppi

**Affiliations:** 1 Department of Chemistry, Life Sciences, and Environmental Sustainability, University of Parma, Parco Area delle Scienze 11/a, 43124 Parma, Italy; 2 Department of Life Sciences, University of Siena, Via PA Mattioli 4, I-53100 Siena, Italy; riccardo.fedeli@student.unisi.it (R.F.); massimo.guarnieri@unisi.it (M.G.); stefano.loppi@unisi.it (S.L.); 3 BAT Center-Interuniversity Center for Studies on Bioinspired Agro-Environmental Technology, University of Naples ‘Federico II’, 80138 Napoli, Italy

**Keywords:** bio-based product, crop resistance, horticultural plants, O_3_, toxic effects mitigation

## Abstract

This study examined whether foliar applications of wood distillate (WD) have a protective effect on photosynthesis and the antioxidant power of lettuce when exposed to an ecologically relevant O_3_ concentration. Seedlings of lettuce (*Lactuca sativa* L.) were fumigated daily with 60 ppb of O_3_ for 30 days, five hours per day. Once per week, 50% of the fumigated plants were treated with foliar applications of 0.2% WD, while control plants were treated with water. The results clearly showed the ability of WD to protect lettuce plants from ozone-induced damage. Specifically, WD-treated plants exhibited lower damage to the photosynthetic machinery, assessed through a series of chlorophyll fluorescence parameters, a higher chlorophyll content, higher antioxidant power, as well as antioxidant molecules, i.e., caffeic acid and quercetin, and higher biomass. Counteracting the overproduction of ozone-generated reactive oxygen species (ROS) is speculated to be the main mechanism by which WD protects the plant from ozone-induced damage.

## 1. Introduction

Ozone (O_3_) is a global strongly oxidizing pollutant, present at ground level following the interaction of UV light with anthropogenic gases, such as carbon monoxide (CO), nitrogen oxides (NO_X_), methane (CH_4_), and non-methane volatile organic compounds (NMVOC_S_) (EPA 2021). Since the mid-20th century, global background ozone (O_3_) concentrations have rapidly increased [1] with current average concentrations for the mid-northern latitude of ca. 30–50 ppb [2].

Plants can tolerate and adapt to O_3_ when chronically exposed to concentrations below 20 nL/L (ca. 21 ppb; [3]). However, when ground-level exceeds the tolerance threshold generally estimated at ca. 40 ppb [4,5], O_3_ becomes phytotoxic and capable of damaging agricultural plants and forest vegetation, and even the biodiversity of a whole ecosystem [2,6]. Following the global increase in O_3_ background concentrations that occurred in the last 20–30 years, 2–15% reductions in the global crop yield were estimated, with a remarkable economic loss [7].

In recent years, the search for solutions to protect crop plants from O_3_ injury has become of global interest, and among the tested methodologies, i.e., the development of O_3_-resistant crops, the selection of resistant germplasm, the use of antiozonants and nanomaterials [8], the use of antioxidants (vitamins, phytohormones, flavonoids, and polyamines), is a very promising strategy to protect crop plants from O_3_ phytotoxicity [9,10,11,12].

Wood distillate (WD), also known as pyroligneous acid or wood vinegar, is a bio-based liquid product obtained from the distillation of the gases produced during the pyrolysis of woody biomass for the production of green energy [13]. Such a product, still unexploited in many agricultural sectors (especially those not involved in organic farming), is now having great success in agriculture as a phytostimulant for crop plants since its use can enhance both plant productivity and endogenous defenses against pathogens [14]. WD can be produced by different feedstocks but the common feature of all WDs is the presence, to a greater or lesser extent, of antioxidant molecules such as phenols [15]. Results from a study conducted on the horticultural plant *Lactuca sativa* L. revealed that foliar application of 0.2% WD deriving from sweet chestnut (*Castanea sativa* Mill.) enhances the content of chlorophyll and biomass of this model species [16], probably owing to its high content of antioxidant compounds, such as polyphenols [17]. Foliar application of WD is also used to increase the defenses of olive (*Olea europaea* L.) trees and grapevine (*Vitis vinifera* L.) plants against pathogens [18,19], but the effectiveness of WD in contrasting the phytotoxic action of O_3_ on crop plant metabolism is still unexplored.

For this reason, this study examines whether foliar applications of WD have a protective effect on lettuce exposed to an ecologically relevant O_3_ concentration.

In detail, we tested if lettuce plants exposed to O_3_ but not treated with WD have a similar response, in terms of photosynthetic system, antioxidants, and yield, as plants treated with WD.

## 2. Materials and Methods

### 2.1. Experimental

Seedlings of lettuce (*Lactuca sativa* L.) cv. Cappuccio bionda were bought from a local nursery. In the laboratory, plants were carefully removed from their growth phytocells and transplanted inside plastic pots (10 × 10 × 12 cm) using a commercial potting soil as the substrate. Seedlings were then acclimatized for one week in a climatic chamber at 20 °C, 70% RH, and 350 μM s^−1^ m^−2^ PAR with 15 h of photoperiod (from 5:00 a.m. to 20:00 p.m.). The whole pool of seedlings (24 plants) was then fumigated daily with 60 ppb of O_3_ for 5 h per day (from 12:00 to 17:00). Once per week, 50% of the fumigated plants (12 plants) were treated with foliar applications of 0.2% sweet chestnut (*Castanea sativa* Mill.) WD (Biodea^®^), while the remaining 50% of plants were sprayed with water and used as a positive control. Foliar applications were run after the light cycle, following the method described by Vannini et al. [16]. All plants were randomly rotated every three days to minimize any possible influence of microclimatic conditions inside the climatic chamber. The experiment lasted four weeks and was replicated three times. The sweet chestnut WD was selected because it had previously been investigated for its safety, for both the environment and humans [17,20,21].

### 2.2. Photosynthetic Parameters

Since photosynthesis is considered the main target of O_3_ phytotoxicity [22], its functionality was assessed by means of selected photosynthetic indicators: the chlorophyll fluorescence, the analysis of the fluorescence transients plotted on a logarithmic scale (OJIP transients), and the chlorophyll content.

#### 2.2.1. Chlorophyll Fluorescence Analyses

The analysis of the chlorophyll fluorescence and the analysis of the OJIP transients are key methodologies to assess the functionality of the photosynthetic machinery following exposure to O_3_ [5,22]. The former was assessed through a number of the OJIP step fluorescence parameters, summarized in Table 1, which describes the ability of the photosynthetic system to absorb, trap, transmit, and convert the absorbed light into energy for CO_2_ fixation [23], while the latter analyzed the ΔV_OP_ profile, calculated by the difference between the V_OP_ profile of treated (Ozone + WD) and control (Ozone) samples (ΔV_OP_ = V_OP_treated (ozone + WD) − V_OP_control(ozone); [24]). Prior to analysis, the seedlings were dark-adapted for 30 min under a dim green light (10 μmol photons m^−2^ s^−1^) and then lighted with an actinic light (3000 μmol photons m^−2^ s^−1^) for one second. Fluorescence analysis was run using a plant efficiency analyzer (Handy PEA, Hansatech Ltd., Norfolk, UK). Fifteen measurements were taken for each replicate.

#### 2.2.2. Analysis of the Chlorophyll Content

The content of chlorophyll was measured by means of a chlorophyll content meter (CCM 300, Opti-Science Inc., Hudson, NH, USA), a non-destructive instrument that quantifies the amount of chlorophyll on a surface basis (mg/m^2^) [25]. Fifteen measurements were taken for each replicate.

### 2.3. Expression of Antioxidants

#### 2.3.1. Total Antioxidant Power

Fresh samples (ca. 200 mg) were homogenized with 4 mL of 80% ethanol and then centrifuged at 15,000 rpm for five minutes. The supernatant (100 µL) was then added to 1 mL of a DPPH solution prepared following the protocol reported by Vannini et al. [26]. After the reaction (ca. 1 h) samples were read at 517 nm and the results were expressed as % Antiradical Activity (ARA%) following the Formulae:

(1) ARA% =100×[1−(sample absorbance/control absorbance)]

where control absorbance is the absorbance of the reagents only.

#### 2.3.2. Content of Caffeic Acid and Quercetin

Given the high number of antioxidant compounds in plant leaves, caffeic acid and quercetin were selected as indicators of polyphenols and flavonoids expression [27,28], respectively. The extraction of caffeic acid and quercetin from lettuce leaves was carried out according to Tokusoglu et al. [29], with modifications. The upper part of each of the major fresh leaves (ca. 1 g) was extracted with 3 mL of 70% acetone containing 1% HCl (*v/v*). After the homogenization, 0.6 mL of pure HCl was added and the final mixture was first vigorously shaken and then left at 90 °C for two hours. Subsequently, samples were shaken and then filtered at 0.45 µm. The extracts were directly analyzed by HPLC (Perkin-Elmer series 200) coupled with a Diode Array Detector (DAD). The analysis of both caffeic acid and quercetin was run according to the method used by Kumar et al. [30], combining water (solvent A) and acetonitrile (solvent B) eluted for 21 min as a mobile phase following the gradient: 0–5 min (80% A), 5–8 min (60% A), 8–12 min (50% A), 12–17 min (40% A), 17–21 min (20% A); an Agilent C18 column (4.6 x 250 mm; particle size 5µm) was used. Runs were monitored at 280 and 325 nm for caffeic acid and quercetin, respectively. Quantifications were carried out using calibration curves of caffeic acid and quercetin (from 5–100 µg/mL), prepared by dissolving the two pure reagents (Sigma, Sant Louis, MI, USA) in the same solvent used to extract both molecules from the samples.

### 2.4. Edible Fresh Biomass

From each plant, visibly undamaged leaves (marketable, i.e., those without necrotic and senescent areas) were removed from shoots and then weighed on a precision balance. Results were expressed as grams on a fresh weight basis (g FW).

### 2.5. Statistical Analysis

To disentangle differences between treated (Ozone + WD) and untreated (Ozone) plants in terms of chlorophyll fluorescence and chlorophyll content, a linear mixed-effect model (LMEM) was fitted for each variable, with treatment as a fixed effect and plant as a random effect [31]. For model validation, scatterplots of the residual and fitted values were used to check for homoscedasticity, and normal probability (qqnorm) plots as well as the Shapiro–Wilk test to check for normality. Models were fitted using the restricted maximum likelihood (REML) estimation, and the significance of the models was checked with type III Anova using the Satterthwaite method [32]. For all the other parameters analyzed, a permutation t-test was used to check for differences between treated (Ozone + WD) and untreated (Ozone) plants. All calculations were run using the free R software [33]; the packages ‘lme4’ and ‘RVAideMemoire’ were used.”

## 3. Results

**Figure 1:**
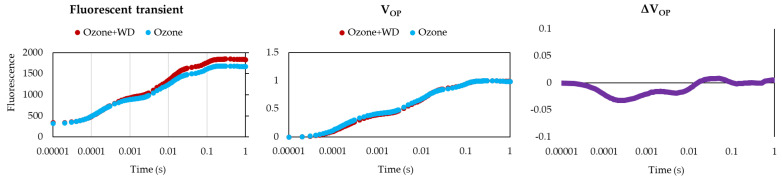
Average log-probit OJIP fluorescence curves and V_OP_ analysis of samples of *Lactuca sativa* after 30 days of fumigation with 60 ppb of O_3_ in combination with (Ozone + WD) or without (Ozone) weekly foliar applications of 0.2% chestnut wood distillate (WD).

**Figure 2:**
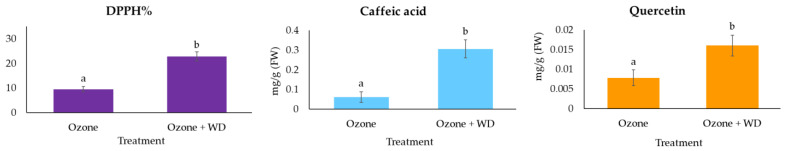
Expression of the antioxidant power (DPPH%) and concentration (mean ± standard error) of both caffeic acid and quercetin in samples of *Lactuca sativa* after 30 days of fumigation with 60 ppb of O_3_ in combination with (Ozone + WD) or without (Ozone) weekly foliar applications of 0.2% chestnut wood distillate (WD). Different letters indicate statistically significant (*p* < 0.05) differences between treatments.

**Figure 3:**
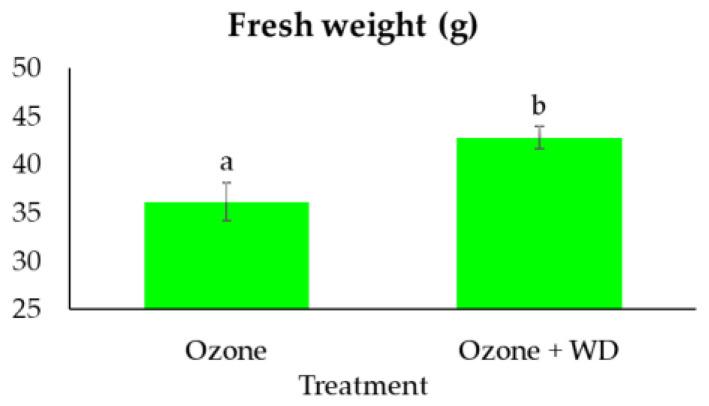
Fresh weight (mean ± standard error) of samples of *Lactuca sativa* after 30 days of fumigation with 60 ppb of O_3_ in combination with (Ozone + WD) or without (Ozone) weekly foliar applications of 0.2% chestnut wood distillate. Different letters indicate statistically significant differences between treatments (*p*-value < 0.05).

WD-treated samples experienced better photosynthetic performances than those only fumigated with O_3_ (Table 2). Specifically, WD-treated samples showed a higher absorbance for excited cross-section (ABS/CS_0_; ca. 3%), energy transmission (TR_0_/CS_0_; ca. 4%), electron transport (ET_0_/CS_0_; ca. 8%), number of reaction centers (RC/CS_0_; ca. 11%), as well as a lower energy dissipation (DI_0_/CS_0_; ca. 4%). Additionally, WD-treated plants showed a higher (*p* < 0.05) expression of the photosynthetic efficiency (F_V_/F_M_; ca. 1%), of the performance index (PI_Abs_; ca. 25%), and a higher content of chlorophyll (ca. 13%). Analysis of the two fluorescence profiles confirmed the results obtained by F_V_/F_M_, while those of the V_OP_ test indicated only negligible differences between treatments (Figure 1). Moreover, samples treated with WD presented a higher (*p* < 0.05) expression of the total antioxidant power (ca. 140%), as well as of both caffeic acid (ca. 400%) and quercetin (ca. 105%) (Figure 2). A higher (*p* < 0.05) biomass (+18%) was also noted (Figure 3).

## 4. Discussion

Lettuce is an ozone-sensitive horticultural plant [34] and O_3_ fumigations caused a decrease in photosynthesis, chlorophyll content, and biomass of this species, consistently with other studies [35,36]. However, when 0.2% WD was sprayed on the leaves, plants showed an improvement in the photosynthetic system, a higher content of chlorophyll and antioxidant molecules, as well as higher biomass, thus suggesting the ability of WD to significantly alleviate the O_3_-induced damage.

WD is rich in antioxidants, such as polyphenols [15,17,37], and is effectively used to increase both the chlorophyll content and biomass of crop plants, as found in some recent studies [16,38,39,40,41]. Although the mechanisms behind its effectiveness are still in need of investigation, it has been suggested that this stimulant effect on plant productivity may be due to the action of antioxidant molecules on cell division [16], in response to the activation of specific transcription genes, as previously observed by Tanase et al. [42,43].

To the best of our knowledge, information on the exact mechanism of action of WD against the O_3_-induced damage is unknown but it is possible that antioxidant molecules play a very important role. We speculate that the pool of antioxidants could have acted in two ways: buffering the interaction between O_3_ and the apoplast, thus reducing the overproduction of ROS, i.e., the main factor responsible for the O_3_-induced toxicity in plants [44], and/or stimulating the synthesis of ROS scavenger molecules, such as superoxide dismutase, peroxidase, and catalase, suggested by Wang et al. [45]. In the first case, WD may have acted as a strengthener of the plant’s antioxidant defenses, while in the second case it could have acted as a stimulator. However, information on which of these two processes plays the greater role has not yet been investigated.

Plants can naturally counteract the oxidative action of O_3_ through the synthesis of antioxidants, such as reduced glutathione [46], but when insufficient or when the oxidative stress is too high, plants may experience reductions in O_3_-sensitive antioxidant molecules, such as caffeic acid and quercetin [47,48,49]. However, following the application of 0.2% WD, our lettuce plants showed a higher content of these molecules compared to the untreated ones, thus suggesting a protective role of WD in preventing the oxidation of antioxidant molecules devoted to counteracting the oxidative pressure induced by O_3_. Consistently with this assumption, WD-treated plants experienced higher antiradical scavenging activity (DPPH) than O_3_-fumigated plants. From a nutritional point of view, both caffeic acid and quercetin have antioxidant, anti-inflammatory, and anticarcinogenic activities [50,51] and in view of the protective action on these molecules from oxidative degradation, WD can be considered not only as a tool to increase plant productivity but also to defend its nutritional content from the oxidative stress. However, caffeic acid showed higher reductions than quercetin, probably because this molecule is involved in several antioxidative roles. It is reported that this compound is also deputed to shield the photosynthetic system from oxidative stresses [52], thus explaining its higher consumption in O_3_-fumigated plants.

Photosynthesis is the main target of O_3_ phytotoxicity [22] and following its damage, both energy transmission mechanisms, reallocation of nutrients, and plant growth can be significantly impaired [44]. Although the photosynthetic system is naturally provided with antioxidant molecules—i.e., caffeic acid—the concentration of ROS above the threshold of the system’s antioxidant defenses may damage it, and programmed cell death processes can be activated [53,54].

Ozone-induced ROS can affect photosynthesis by either damaging structurally the chloroplast, as evident by ultrastructural changes [26,55], and/or activating cascade signals which lead to stomatal closure and, in turn, generate further reductions in net photosynthesis, CO_2_ assimilation, and plant growth [2,44]. As a result, however, reductions in carbon assimilation may induce increases in the electron transfer with the consequent increase in unwanted ROS overproduction [56]. Hence, the photosynthetic system starts a self-regulatory process to achieve protection from photo-oxidation, leading to an immediate reduction in both the content of chlorophyll and the number of reaction centers [22,57,58], i.e., the structures dedicated to intercepting light to energetically supply the photosynthetic system [59]. With this process, dissipation (DI_0_) at the expense of absorption and transmission (TR_0_ and ET_0_) is favored [22] and reductions in the activity of both PSI and PSII (measured as F_V_/F_M_ and PI) can be measured, as observed for Canola (*Brassica napus* L.) and tomato (*Solanum lycopersicum* L.) plants in open top-chamber fumigations [5,60]. Nevertheless, following WD foliar applications, all the above-mentioned negative effects on the electron transport chain of the photosynthetic system were significantly alleviated. In fact, WD-treated plants experienced a higher number of active reaction centers (RC), positive values for the parameters involved in the energy absorption, trapping, and transmission from the PSII to PSI (ABS, TR, ET), as well as lower heat dissipation energy (DI). Additionally, WD-treated plants showed also a higher expression of the photosynthetic efficiency F_V_/F_M_, the maximum quantum yield of primary photochemistry, the performance index PI, the overall indicator of the PSI and PSII functionality [61], and higher content of chlorophyll. Since RCs are naturally subjected to oxidative stress forcing them to be rebuilt very quickly [53], we speculate that WD, thanks to its content of antioxidants, may have greatly reduced this turnover or increased its frequency. Higher photosynthetic efficiency also means more biomass, with a 10% increase in the ratio for every 30% increase in photosynthesis [62]. Ozone can effectively reduce photosynthesis with inevitable consequences for plant growth [44], but by shielding the photosynthetic system from oxidation, the plant’s growth and biomass can also be protected.

## 5. Conclusions

The results of the present study clearly showed the ability of WD to protect lettuce plants from ozone-induced damage. Specifically, WD-treated plants exhibited lower damage to the photosynthetic machinery, a higher content of chlorophyll (ca. 13%), a higher antioxidant power (ca. 140%), as well as antioxidant molecules (i.e., caffeic acid and quercetin, ca. 400 and 105%, respectively), and higher biomass (ca. 18%). Counteracting the oxidative stress that occurred at the level of the photosynthetic system is speculated to be the main mechanism by which WD protects the plant from ozone-induced damage. Moving forward, WD can be seen not only as a natural product for stimulating plant productivity but also as a means of protecting crop plants against oxidative stress.

## Figures and Tables

**Table 1:** Description of the measured photosynthetic parameters.

Parameter	Description
ABS/CS_0_	Absorbance for excited cross-section
TR_0_/CS_0_	Trapping flux for excited cross-section
ET_0_/CS_0_	Energy transmission for excited cross-section
RC/CS_0_	Number of reaction centers for excited cross-section
DI_0_/CS_0_	Heat dissipation for excited cross-section
F_V_/F_M_	Photosynthetic efficiency
PI_ABS_	Performance index

**Table 2:** Expression of photosynthetic parameters (mean ± standard error) in samples of *Lactuca sativa* after 30 days of fumigation with 60 ppb of O_3_ in combination with (Ozone + WD) or without (Ozone) weekly foliar applications of 0.2% chestnut wood distillate (WD).

Parameter	Ozone	Ozone + WD	*p*-Value
ABS/CS_0_	293 ± 2.5 a	301 ± 2.6 b	*p* < 0.05
DI_0_/CS_0_	51 ± 0.5 a	49 ± 0.5 b	*p* < 0.01
TR_0_/CS_0_	242 ± 2.0 a	252 ± 2.1 b	*p* < 0.001
ET_0_/CS_0_	131 ± 1.4 a	141 ± 1.4 b	*p* < 0.001
RC/CS_0_	105 ± 1.1 a	117 ± 1.1 b	*p* < 0.001
F_V_/F_M_	0.826 ± 0.001 a	0.837 ± 0.001 b	*p* < 0.001
PI_ABS_	2.05 ± 0.03 a	2.56 ± 0.04 b	*p* < 0.001
Chlorophyll (mg/m^2^)	200 ± 3.7 a	225 ± 2.7 b	*p* < 0.001

Different letters indicate statistically significant (*p* < 0.05) differences between treatments. Abbreviations: absorbance for excited cross-section (ABS/CS_0_), heat dissipation for excited cross-section (DI_0_/CS_0_), trapping flux for excited cross-section (TR_0_/CS_0_), energy transmission for excited cross-section (ET_0_/CS_0_), number of reaction centers for excited cross-section (RC/CS_0_), photosynthetic efficiency (F_V_/F_M_), performance index (PI_ABS_), chlorophyll content (Chlorophyll).

## Data Availability

The raw data presented in this study are available on request from the corresponding author.
